# Correction: FastKAN-DDD: A novel fast Kolmogorov-Arnold network-based approach for driver drowsiness detection optimized for TinyML deployment

**DOI:** 10.1371/journal.pone.0340596

**Published:** 2026-01-06

**Authors:** Siham Essahraui, Ismail Lamaakal, Yassine Maleh, Khalid El Makkaoui, Mouncef Filali Bouami, Ibrahim Ouahbi, Hela Elmannai, Ahmed A. Abd El-Latif

There are errors in the Acknowledgement statement. The correct Acknowledgement statement is as follows: Princess Nourah bint Abdulrahman University Researchers Supporting Project number (PNURSP2025R747), Princess Nourah bint Abdulrahman University, Riyadh, Saudi Arabia.

There is an error in affiliation 3 for author Hela Elmannai. The correct affiliation 3 is: Department of Information Technology, College of Computer and Information Sciences, Princess Nourah bint Abdulrahman University, P.O. Box 84428, Riyadh 11671, Saudi Arabia.
